# Consistent declines in wing lengths of Calidridine sandpipers suggest a rapid morphometric response to environmental change

**DOI:** 10.1371/journal.pone.0213930

**Published:** 2019-04-03

**Authors:** Alexandra M. Anderson, Christian Friis, Cheri L. Gratto-Trevor, R. I. Guy Morrison, Paul A. Smith, Erica Nol

**Affiliations:** 1 Trent University, Environmental and Life Sciences, Peterborough, Ontario, Canada; 2 Canadian Wildlife Service, Environment and Climate Change Canada, Toronto, Ontario, Canada; 3 Prairie and Northern Wildlife Research Centre, Environment and Climate Change Canada, Saskatoon, Saskatchewan, Canada; 4 National Wildlife Research Centre, Environment and Climate Change Canada, Ottawa, Ontario, Canada; 5 Trent University, Biology Department, Peterborough, Ontario, Canada; University of Pavia, ITALY

## Abstract

A recent study demonstrated that semipalmated sandpiper (*Calidris pusilla*) wing lengths have shortened from the 1980s to the present-day. We examined alternative and untested hypotheses for this change at an important stopover site, James Bay, Ontario, Canada. We evaluated morphometric patterns in wing length and bill length by age and sex, when possible, and assessed if wing shape has also changed during this time-period. We investigated patterns of morphological change in two additional Calidridine sandpipers, white-rumped sandpipers (*Calidris fuscicollis*) and least sandpipers (*Calidris minutilla*), to determine if shorter wing lengths are a widespread pattern in small sandpipers. We also examined allometric changes in wing and bill lengths to clarify if wing length declines were consistent with historical scaling relationships and indicative of a change in body size instead of only wing length change. We found that including sex and wing shape in analyses revealed important patterns in morphometric change for semipalmated sandpipers. Wing lengths declined for both sexes, but the magnitude of decline was smaller and not significant for males. Additionally, semipalmated sandpiper wings have become more convex, a shape that increases maneuverability in flight. Wing lengths, but not bill lengths, declined for most species and age classes, a pattern that was inconsistent with historical allometric scaling relationships. For juvenile semipalmated sandpipers, however, both bill and wing lengths declined according to historical scaling relationships, which could be a consequence of nutritional stress during development or a shift in the proportion of birds from smaller-sized, western breeding populations. Except for juvenile semipalmated sandpipers, we did not find evidence for an increase in the proportion of birds from different breeding populations at the stopover site. Given the wide, hemispheric distribution of these sandpipers throughout their annual cycles, our results, paired with those from a previous study, provide evidence for wide-spread reduction in wing lengths of Calidridine sandpipers since the 1980s. The shorter wing lengths and more convex wing shapes found in this study support the hypothesis that selection has favored more maneuverable wing morphology in small sandpipers.

## Introduction

Persistence of wildlife populations depends on suitable responses of individuals to environmental change. Changes in phenology [1], distribution [2], population size [3], and morphology [4] have been documented across many taxa in response to changing environmental conditions. Understanding how environmental change has affected organisms in the past will be useful for predicting the future viability of wildlife populations.

Morphological change in flora and fauna is a widespread response to environmental change and can occur through phenotypic plasticity and/or natural selection [5–7]. Although change in the bill morphology of Darwin’s finches after drought is the best-known example [8–10], morphometrics also can change as a result of climate change [4], [11–16], urbanization [12], [17]-[19], and predation risk [20–22].

Smaller body size is a predicted morphological response to increasing global temperatures and variable precipitation associated with climate change because of direct (i.e., increased total metabolic rate and respiration) or indirect costs (i.e., changes in prey, water, or nutrient availability) [4], though see Gardner et al. [23]. A recent example of this comes from red knots (*Calidris canutus canutus*). Red knot chicks that hatched in years with earlier snow melt in the Arctic were smaller than birds hatched in years when snow melted later, possibly a result of a mismatch in phenology of red knot and their prey, coupled with a decline in abundance and size of available invertebrate prey [15]. In addition to climate change, morphological change can also result from pressures such as changes in predation risk or human disturbance. For example, over time, wings of cliff swallows (*Petrochelidon pyrrhonota*) became shorter because short-winged swallows were less likely to be killed by vehicles [18], and hindwing shape of damselflies (*Calopteryx splendens*) became more maneuverable in locations with higher predation by an avian predator [22].

In birds, morphological changes in wing size and shape are common [13], [18], [24], [25] and important because they have consequences for flight mechanics, including long-distance flight efficiency, predator evasion, and breeding displays, all of which can affect fitness. In general, round, short wings increase agility and maneuverability, which aids in predator avoidance [26] and/or acrobatic breeding displays by facilitating tighter turns and rolls [27]. Long, pointed wings, on the other hand, are more efficient for sustained flights such as migration [28]-[30]. Hence competing pressures influence wing morphology in birds.

Calidridine sandpipers are a group of birds that may experience competing selective pressures for wing size and shape. Sandpipers in the genus *Calidris* have long, pointed wings that facilitate efficient long-distance migrations [31–33]. As small birds, sandpipers also often fall victim to predation by raptors [34–36], and predation pressure by raptors could favour more maneuverable wing shapes [37]. A recent study documented wing length declines in semipalmated sandpipers (*Calidris pusilla*) across their breeding range and at stopover sites in North America [25]. The authors hypothesized that shorter wings occurred because increased predation risk from the recovery of peregrine falcons (*Falco peregrinus*) after the ban of DDT in the early 1970s selected for more maneuverable wings. The study, though covering wide temporal and spatial scales, did not examine alternative hypotheses such as the impacts of age or sex differences on wing length declines. Wings of young birds tend to be shorter and rounder than wings of adults [38], [39], potentially as an antipredator adaptation [38]. Additionally, sexual dimorphism in wing length is widespread in shorebirds [40], [41]. In the genus *Calidris*, males tend to be smaller than females [40], but the sexes overlap in size so molecular methods are necessary to identify sex [42], [43]. Therefore, it is possible that wing length declines observed by Lank et al. [25] were the result of an increase in the proportion or length of stay of birds with shorter wings (males or juveniles).

In this work, we examine alternative hypotheses for changes in wing morphology of three species of Calidridine sandpipers at an important shorebird migratory stopover site. We used banding data (historical: 1974–1982 [44] and present-day: 2014–2017) to assess morphological change for semipalmated sandpipers (SESA), least sandpipers (LESA, *Calidris minutilla*), and white-rumped sandpipers (WRSA, *Calidris fuscicollis*). We determined if wing lengths are shorter in present-day birds for the three *Calidris* species, across age classes and sex, where possible. Additionally, we tested whether wing length declines were a result of 1) apparent changes (subpopulation shifts, age or sex ratio changes, increase in feather wear) or 2) true morphometric changes (a reduction in overall body size, wing size, or change in wing shape).

### Examining hypotheses of morphometric change

#### Predictions for apparent morphometric change

Apparent wing length change could result from different breeding subpopulations congregating at James Bay in different proportions than they did historically. Geographic variation in morphology for breeding sandpiper subpopulations may aid in determining changes in the proportion of birds arriving in James Bay from specific Arctic and subarctic breeding areas. Semipalmated sandpipers vary in size longitudinally across North America; birds in the west have shorter wings and bills than birds in the east [44], [45]. White-rumped sandpipers, on the other hand, show latitudinal variation in morphology; birds at the northern-most breeding sites have longer wings and bills than those at the southern-most breeding sites [46]. If the relative abundance of birds from different regional subpopulations has changed at James Bay, wing and bill lengths for semipalmated and white-rumped sandpipers will increase or decrease together. We could not test this hypothesis for least sandpipers because data are limited, and geographic variation in wing and bill lengths is not consistent across the sampled breeding areas [47].

If declines in wing lengths are driven by age or changing age ratios, we will see differences in wing length between adults (> 1 year old) and juveniles (< 1 year old) and either a decline in wing lengths of only one of the age classes or a change in age ratios in favor of the shorter-winged age class. We test this hypothesis using data from semipalmated sandpipers because both age classes use southwestern James Bay as a stopover site, whereas few white-rumped sandpiper juveniles or least sandpiper adults are observed at the site.

Changes in wing length by sex were tested using a dataset from sexed semipalmated sandpipers. If wing length declines arise from changing sex ratios, then wing lengths of each sex will not have changed over time; instead, an increase in the proportion of males (shorter wings) could result in apparent wing length declines overall. Due to differential patterns in migration timing by sex in these sandpipers [48], [49], changes in sex ratios should result in a seasonal pattern of shorter wing lengths during the time-period when females tend to migrate through the stopover area but not during the time when males tend to migrate. For example, for least sandpipers [48], [49] and semipalmated sandpipers [48], shorter wings would occur earlier in the fall when females tend to migrate but not late in the fall when most males tend to move through. For white-rumped sandpipers, differences in migration phenology by sex are unclear; however, males do not care for young in this species and may leave breeding areas earlier than females [50], so we would expect to see the opposite pattern to that observed in the other species.

Feather wear from feather age [51] or increased flight demands such as longer migratory routes [52] could lead to apparent wing length change. If wings have shortened from increased feather wear, we expect to see declines in wing lengths for adults that grew their primaries during the previous winter and have subsequently undergone a northbound migration and breeding season. In contrast, wing lengths of juveniles who have just grown their primary feathers weeks before arriving at James Bay will not have shortened.

#### Predictions for true morphometric change

If birds are getting smaller overall (for example as a result of warming climate), we predict that both wing and bill lengths of sandpipers will shorten over the 40-year study period across species and age classes following historical allometric scaling relationships. This pattern is expected because wing and bill lengths are associated with body size (and frequently used in principal components analyses to determine a structural size variable; e.g. [53]). Alternatively, if only wings have shortened, we expect to see shorter wings but no change in bill lengths for all groups.

We examine if shorter wings are paired with increased wing convexity and roundness to determine if wing shape has changed to favor agility and maneuverability, which would support the predation risk hypothesis suggested by Lank et al. [25]. This paper hypothesized that recent shortening of semipalmated sandpiper wing lengths increases sandpiper maneuverability when encountering avian predators. However, if wings are shorter but not rounder and mass is constant, birds would have higher wing loading (mass/wing area), which decreases take-off speed [54], [37] and escape angle [37], [55]. Although high wing loading and rounder wings are energetically costly during migration [30], rounder wings maximize thrust and may increase acceleration and maneuverability to escape aerial predators [29]. Therefore, for shorter wings to increase maneuverability, they must also be rounder.

## Materials and methods

### Field methods

**Fig 1:**
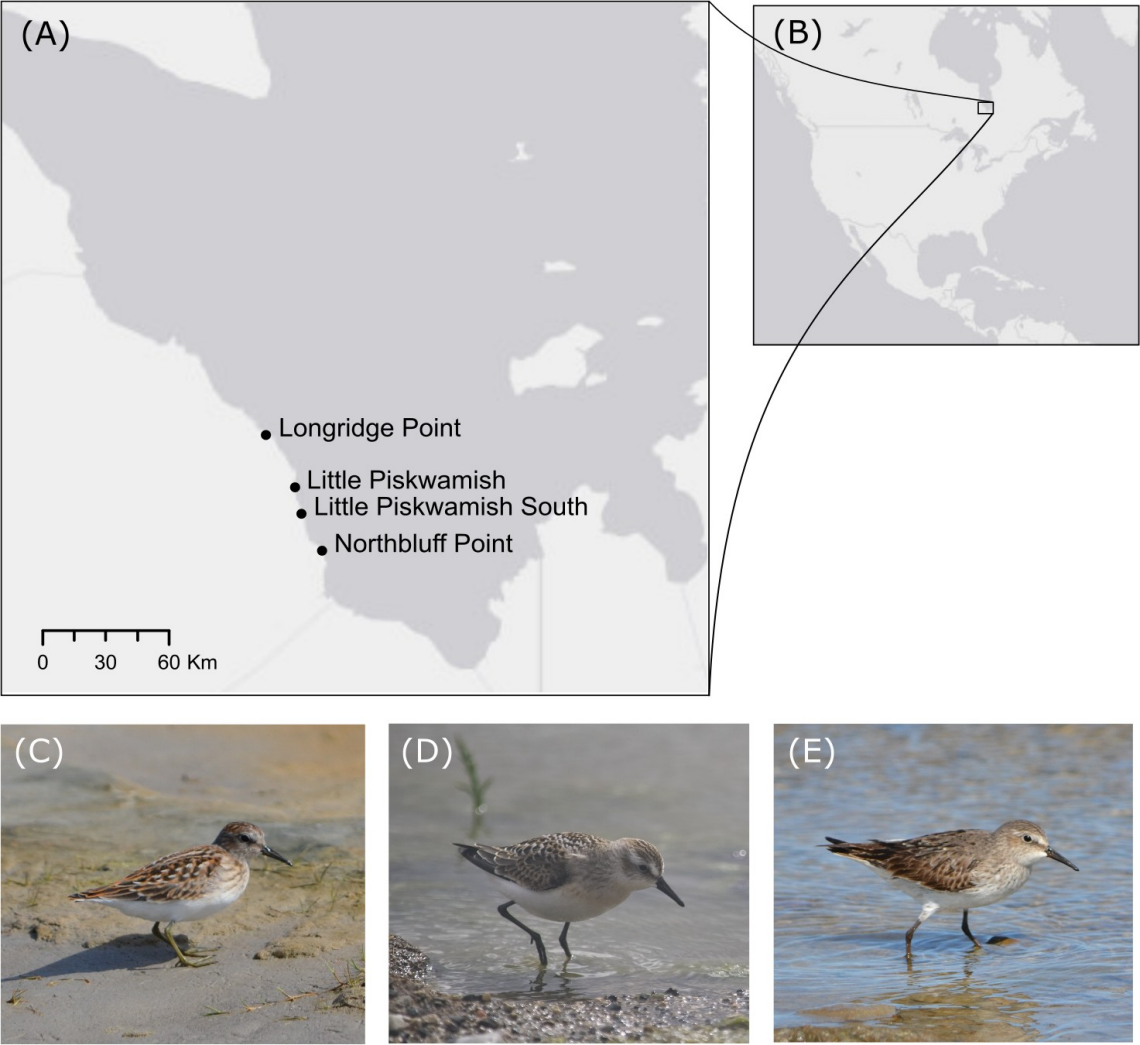
Shorebird banding locations along the southwestern coast of James Bay, Ontario, Canada, and photos of study species. (A) Locations of shorebird banding along the southwestern coast of James Bay. (B) The location of James Bay in North America. (C) A juvenile least sandpiper. (D) A juvenile semipalmated sandpiper. (E) An adult white-rumped sandpiper.

We analyzed wing and bill lengths from historical (1974–1982) and present-day (2014–2017) banding records of least (Fig 1C), semipalmated (Fig 1D), and white-rumped sandpipers (Fig 1E) captured along the southwestern coast of James Bay, Ontario, Canada (Fig 1B) in the Traditional Territory of Moose Cree First Nation, during southbound migration and stopover (approximately July 17/18^th^—September 10/11^th^). Least sandpiper banding data were truncated to July 28^th^/29^th^ through September 1^st^/2^nd^ because historically, least sandpipers were not captured in September and few least sandpipers were captured in mid-July during present-day banding operations. Mass was not used as a measure of body size because at this stopover site, mass is variable, and birds can double their mass to support long-distance migratory flights [56]; therefore, it is not a reliable measure of structural size. Shorebirds were captured both day and night with mist nets located on the intertidal flats at one to four remote field camps per year (Northbluff Point, 51.4839°N, -80.4517°W; Longridge Point, 51.7986°N, -80.6915°W; Little Piskwamish Point, 51.6578°N, -80.5675°W; and Little Piskwamish South, 51.5851°N, -80.5386°W; Fig 1A).

Measurements of maximum flattened wing length (± 1 mm, ruler) and culmen length (± 0.1 mm, calipers) from live birds were taken in all years using the methods described in The North American Bander’s Manual for Banding Shorebirds [57]. Birds were aged by plumage as hatch-year (hereafter juvenile) or after-hatch-year (hereafter adult), based on the shape and color of median wing coverts [57]. Birds older than one year were classified as adults because in these species, of the few yearlings that undertake a northbound migration [57]-[59], most undergo a partial postjuvenal wing molt of their outer primaries [57], [58] and are indistinguishable from birds older than two years of age. In both historical and present-day, birds were measured and aged by multiple banders (>15 banders in each time-period). We collected blood samples from a subset of semipalmated sandpipers in the present-day for molecular sexing. Blood (75–150 μL) was collected from the brachial vein of a bird using capillary tubes. Plasma was separated from red blood cells by centrifugation for separate analyses, and red blood cells were stored in 95% ethanol and frozen prior to DNA extraction.

Birds were released after banding, and Animal Care Committees from Trent University (Protocol 23904) and Environment and Climate Change Canada (Protocol 14CF01, 15CF01, 16CF01, 17CF01) approved all present-day bird capture, handing, and blood sampling methods. Migratory birds are protected under the Migratory Bird Convention Act in Canada; therefore, bird capture methods and blood samples were collected under permit from Environment and Climate Change Canada. All research was conducted on provincial land within the Traditional Territory of Moose Cree First Nation, and permission was received from the Ontario Ministry of Natural Resources and Forestry and Moose Cree First Nation Lands and Resources. Private owners provided permission for field crews to live at hunt camps while conducting this research.

### Sex determination

Historical data of shorebird sex were only available for semipalmated sandpipers, so we used data from this species to determine if wing lengths have shortened by sex. Sex of semipalmated sandpipers was obtained by dissection of mist net mortalities (historical birds) and molecular sexing of shorebird blood samples (present-day birds). In the historical dataset, wing and bill measurements were taken in the field at the time of death prior to freezing; hence no post-mortem shrinkage of wing or bill lengths is expected for these measurements [60]. For present-day samples, sex was determined from red blood cells using primers and molecular methods described in Van der Velde et al. [61].

### Wing shape determination

Semipalmated sandpiper wing roundness and wing convexity were calculated using size constrained correspondence analysis (SCCA) [29]. This method calculates wing-tip shape indices (wing roundness/pointedness, Component 2 (C2) and wing convexity/concavity from the tip (C3), both of which are independent of body size (C1)). One observer (AMA) measured maximum flattened primary lengths of folded wings from semipalmated sandpiper study skins collected from the southwestern coast of James Bay during the historical study period (1980–1981; housed at the Royal Ontario Museum, Toronto, Ontario, Canada). The longest primary (P10 in most birds in our study) was measured with a ruler (± 1 mm), and the distance between the longest primary and the next seven longest primaries (ΔQ values) [29] were measured using calipers (± 0.1 mm). Historical wing lengths were increased by 1.6% to account for shrinkage of small sandpiper museum specimen wing lengths [60] prior to calculating primary lengths using ΔQ values. The same measurements were recorded on a subset of present-day semipalmated sandpipers in the field (n = 20; measured by AMA). To reduce bird-handling time, photos of folded, flattened wings of live birds were taken with a scale-reference in the frame and measured using ImageJ [62]. Photos were then calibrated (S1 Appendix) using caliper measurements from the subset of measured live birds. New variables of wing roundness and wing convexity from the SCCA were calculated for each bird and used as response variables in linear models with time-period, age, a time-period by age interaction, and day of year as a covariate. SCCA analyses were conducted using R code by Blankers et al. [63].

### Statistical methods

#### Wing and bill models

We analyzed data from juvenile least sandpipers, adult white-rumped sandpipers, and both age classes of semipalmated sandpipers. Only birds for which both bill length and flattened wing length were measured were included in analyses. Data were analyzed using linear models, and separate bill length and wing length models were analyzed for each species. For all models, maximum flattened wing length or bill length was the response variable, and time-period (historical or present-day), day of year, and an interaction between time-period and day of year were included as model predictors. Because individually marked birds move between field camps within a season (personal observation from transmitter data), camp was not included as a variable in the models. A day of year by time-period interaction was included to assess varying seasonal patterns in morphometrics. Models for semipalmated sandpipers also included age, an age by time-period interaction, an age by day of year interaction, and a three-way interaction between age, time-period, and day of year. To determine changes in morphology of semipalmated sandpipers by sex, we ran separate bill and wing length models for the subset of data of sexed birds with sex, age, time-period, day of year, an interaction between sex and time-period, an interaction between time-period and age, and a three-way interaction between sex, time-period, and age as predictors.

#### Wing and bill allometry

We used linear models with bill length as the response variable and maximum flattened wing length, time-period, and an interaction between time-period and wing length as predictors to determine whether the relationship between wing length and bill length has changed over time. Separate models were created for each species and age class, and day of year and an interaction between day of year and time-period were included as model covariates.

#### Measurement error

We assessed inter-observer wing and bill length measurement error to determine if observed differences in wing and bill lengths could be detected reliably with multiple measurers. Error was assessed using historical data from within-year recaptures of individual birds measured by different banders. Present-day banding data were not analyzed for measurement error because individual birds were not recaptured (a result of lower sampling intensity). Measurement error was determined using a linear mixed effects model in the R package lme4 [64] with species as a fixed effect and measurer and individual bird as random factors. The random effect of bird determines the variance in measurements from the same bird between two different measurers. The random effect of measurer accounts for instances when an individual bander measured more than one bird. It, therefore, accounts for an individual bander having some amount of bias (i.e., consistently lower or higher measurement than other observers). Separate models were analyzed for bill length and wing length, and measurement error was estimated as the standard error of the model residual term [65], [66]. The repeatability coefficient for measurement error (CR) between two measurers (the range within which 95% of differences in measurements fall) was calculated following Vaz et al. [66], and repeatability of measurements (*R*) was calculated using the package rptR [67].

Estimated marginal means (i.e., means estimated from models while controlling for other model variables) were calculated using the lsmeans package in R [68]. For all models, assumptions of model fit were checked by viewing diagnostic plots of residuals [69]. We also report and interpret the results of each model as a global model which includes all terms, including terms that were not significant, because non-significant values had biological meaning based on *a priori* hypotheses [70]. This approach is more transparent for hypothesis testing and provides unbiased parameter estimates [70], [71]. All statistical analyses were conducted using R version 3.5.1 [72]. Boxplots were created using the ggplot2 package [73], and wing and bill covariance plots were created in R using the sjPlot package [74].

## Results

We analyzed 43,768 historical banding records and 1,913 present-day banding records (SESA juvenile: historical n = 14,717 and present-day n = 996; SESA adult: historical n = 23,872 and present-day n = 429; LESA juvenile: historical n = 2,526 and present-day n = 266; WRSA adult: historical n = 2,653 and present-day n = 222). A subset of present-day semipalmated sandpipers were sexed (females n = 64, males n = 60) and compared to historical mist net mortalities (females n = 60, males n = 45).

### Wing length models

**Fig 2:**
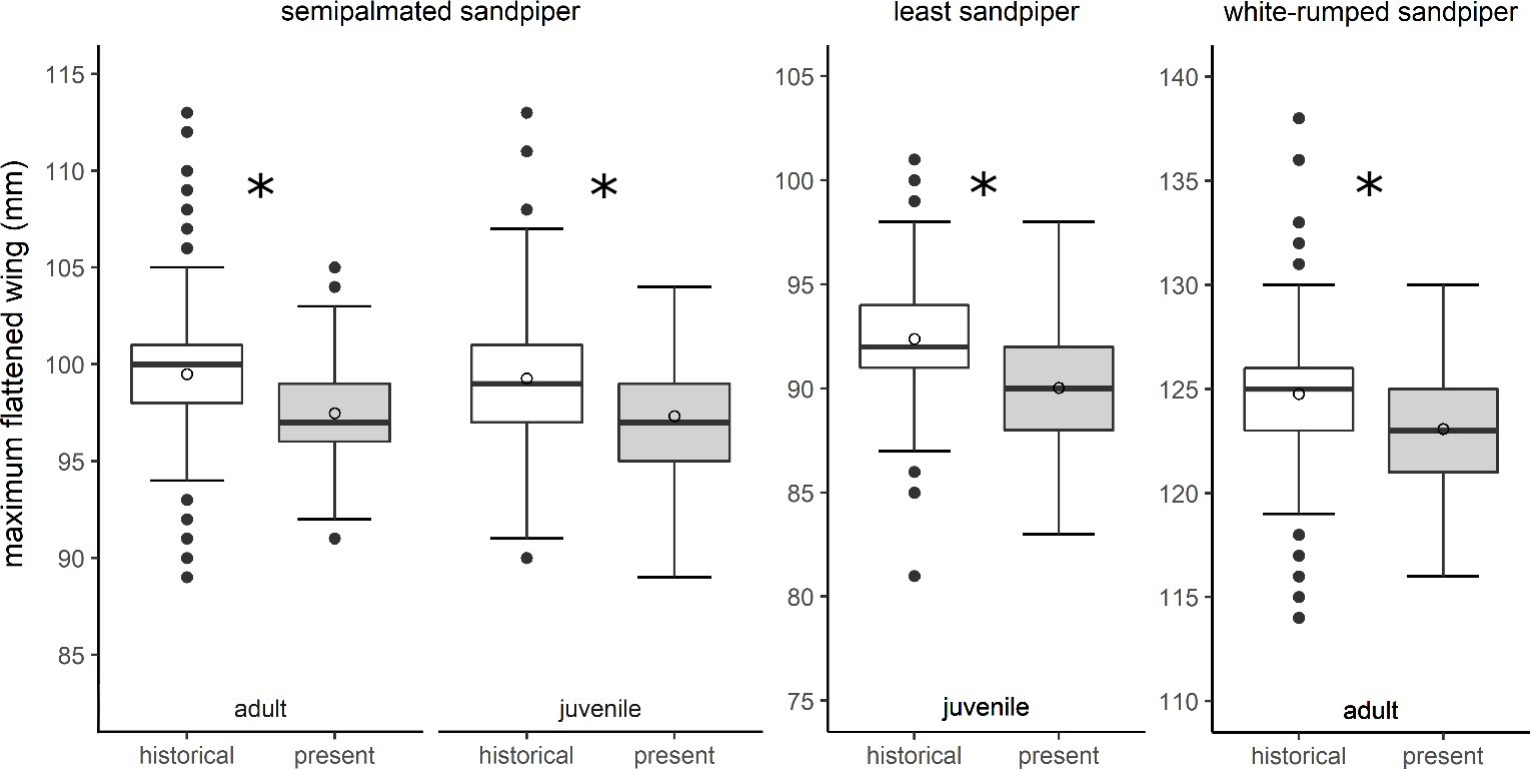
Wing lengths of three sandpiper species are shorter than in the 1980s. Historical (1974–1982) and present-day (2014–2017) differences in wing lengths of semipalmated, least, and white-rumped sandpipers. Significant differences are designated with * (α = 0.05). Open circles represent model predicted least squares means.

Wing lengths declined for all species and age classes (Table 1, Fig 2). Present-day sandpiper wings were approximately 2.0 mm shorter than historical wings, though the magnitude of decline was greatest for least sandpipers and lowest for white-rumped sandpipers (Table 2). For least sandpipers, a seasonal decline in wing length was observed in present-day birds but not in historical birds (significant time-period by day of year interaction). Despite the interaction, present-day least sandpipers had shorter wings throughout the season. Present-day least sandpiper wings were ~1.5 mm shorter than historical wings at the start of the capture period and ~3.5 mm shorter at the end of the capture period. As predicted, wing lengths declined seasonally for semipalmated sandpipers (~1.1 mm over the 56 d capture period for the species) and increased seasonally for white-rumped sandpipers (~1.3 mm over the 49 d capture period). The slopes of these changes did not differ by time-period (interaction not significant).

**Table 1:** Global wing length model results.

	semipalmated sandpiper both ages				least sandpiper juveniles				white-rumped sandpiper adults			
	β	SE	F	*p*	β	SE	F	*p*	β	SE	F	*p*
intercept	103.7	0.4		***<0*.*001***	93.1	1.6		***<0*.*001***	118.6	1.4		***<0*.*001***
present-day	-7.1	3.0	942.7	***<0*.*001***	10.4	4.4	213.5	***<0*.*001***	2.9	4.0	85.3	***<0*.*001***
day of year	-0.02	0.0	157.2	***<0*.*001***	0.0	0.0	2.6	0.11	0.03	0.0	17.9	***<0*.*001***
juveniles	-1.1	0.7	29.9	***<0*.*001***								
present-day by day of year	0.02	0.01	0.1	0.73	-0.1	0.02	8.6	***<0*.*01***	-0.02	0.02	1.3	0.255
present-day by juveniles	6.3	3.6	0.5	0.48								
day of year by juveniles	0.0	0.0	1.0	0.33								
present-day by day of year by juveniles	-0.3	0.02	2.8	0.09								

Results from linear models of wing length with temporal and demographic variables as predictors for three species of sandpipers measured along the southwestern coast of James Bay in two time-periods: 1974–1982 and 2014–2017. Significant *p*-values are italicized and bolded (α = 0.05). Blank cells indicate that the parameter was not included in the model for that species. For semipalmated sandpipers, the model reference group (the intercept coefficient) is adults in the historical time-period. For least and white-rumped sandpipers, the reference group is birds in the historical time-period.

**Table 2:** Changes in wing and bill lengths of sandpipers by age.

		semipalmated sandpiper		least sandpiper	white-rumped sandpiper
		juveniles	adults	juveniles	adults
**wings**	historical	99.3 ± 0.0	99.5 ± 0.0	92.4 ± 0.1	124.8 ± 0.1
	present-day	97.3 ± 0.1	97.5 ± 0.2	90.0 ± 0.2	123.1 ± 0.2
	difference	**↓ 2.0** ± **0.1**	**↓ 2.0** ± **0.2**	**↓ 2.4** ± **0.2**	**↓ 1.7** ± **0.2**
**bills**	historical	19.6 ± 0.0	19.3 ± 0.0	18.5 ± 0.0	23.3 ± 0.0
	present-day	19.0 ± 0.1	19.3 ± 0.1	18.3 ± 0.1	23.2 ± 0.1
	difference	**↓ 0.5** ± **0.1**	0.1 ± 0.1	**↓ 0.2 ± 0.1**	0.1 ± 0.1

Least square means and mean differences for models of wing length (mm) and bill length (mm) ± standard error for three species of sandpipers measured along the southwestern coast of James Bay in two time-periods: historical (1974–1982) and present day (2014–2017). Means were calculated holding covariates constant at their mean. Significant mean differences are bold (α = 0.05), and the direction of the difference is shown.

### Bill length models

**Fig 3:**
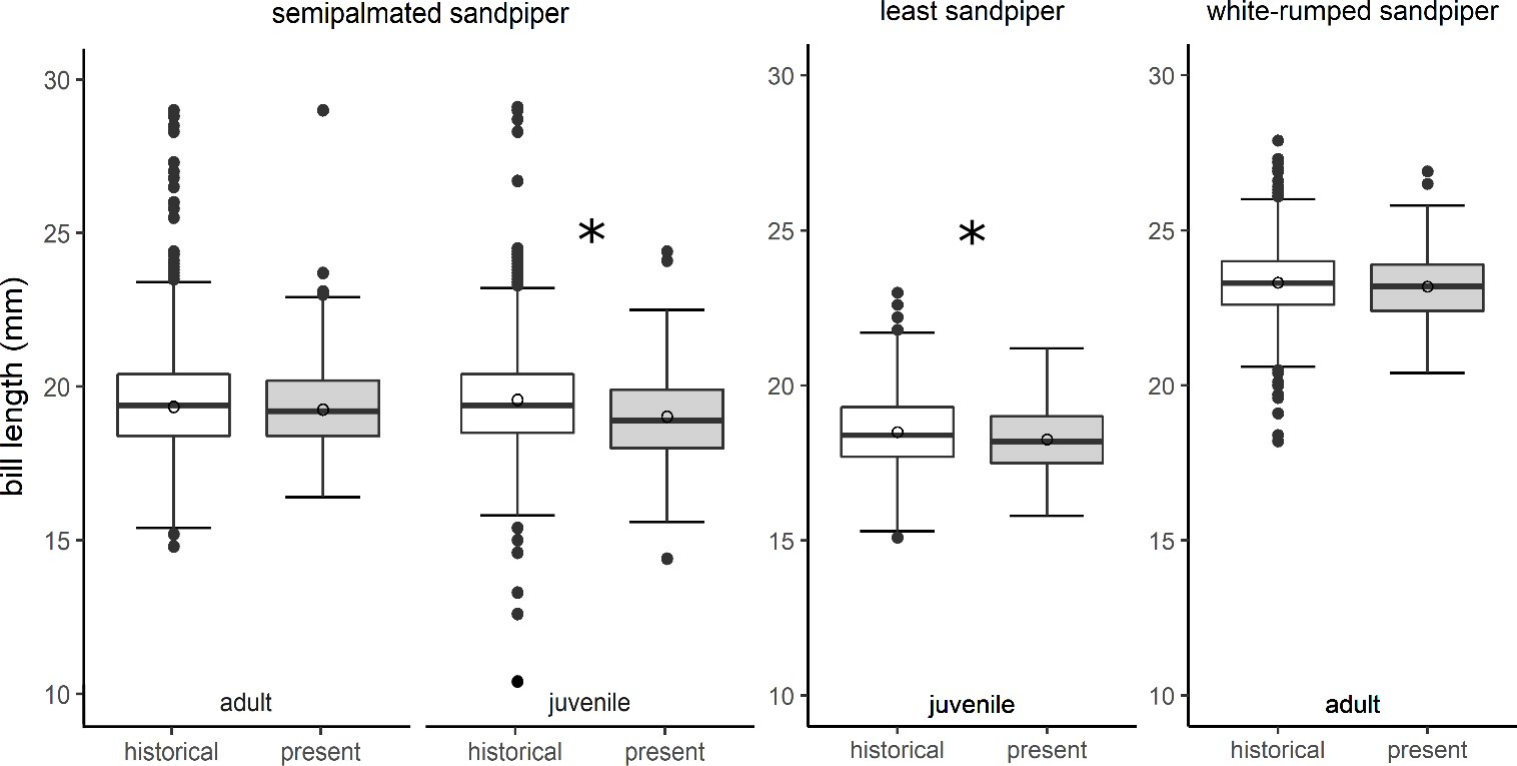
Bill lengths changes of small sandpipers are inconsistent across species and age classes. Historical (1974–1982) and present-day (2014–2017) differences in bill lengths of semipalmated, least, and white-rumped sandpipers. Significant differences are designated with * (α = 0.05). Open circles represent model predicted least squares means.

Compared to changes in wing length, bill lengths did not change consistently across species (global model results in Table 3). Bill lengths declined for semipalmated and least sandpiper juveniles but not for semipalmated or white-rumped sandpiper adults (Fig 3). However, the magnitude of decline for least sandpipers (Table 2) was within the range of inter-observer measurement error for bill lengths (see Measurement error below). Like wing lengths, we detected seasonal patterns in bill length change. For semipalmated sandpipers, bill lengths declined throughout the season, and the slopes of seasonal decline differed by age. Least sandpiper bills also declined seasonally (0.5 mm decline in bill length over the 31 d capture period for the species). White-rumped sandpiper bill lengths increased seasonally (~ 0.8 mm over the 49 d capture period).

**Table 3:** Global bill length model results.

	semipalmated sandpiper both ages				least sandpiper juveniles				white-rumped sandpiper adults			
	β	SE	F	*p*	β	SE	F	*p*	β	SE	F	*p*
intercept	22.7	0.2		*<0*.*001*	22.1	0.7		*<0*.*001*	19.7	0.6		*<0*.*001*
present-day	-0.7	1.6	106.4	***<0*.*001***	1.1	2.1	10.6	***<0*.*01***	-0.01	1.7	2.7	0.10
day of year	-0.02	0.0	338.2	***<0*.*001***	-0.02	0.0	30.5	***<0*.*001***	0.02	0.0	41.2	***<0*.*001***
juveniles	-0.6	0.4	153.4	***<0*.*001***								
present-day by day of year	0.0	0.0	2.0	0.15	-0.01	0.01	0.4	0.51	0.0	0.01	0.0	0. 95
present-day by juveniles	-1.3	1.9	20.2	***<0*.*001***								
day of year by juveniles	0.0	0.0	5.6	***0*.*02***								
present-day by day of year by juveniles	0.0	0.01	0.2	0.68								

Results from linear models of bill length with temporal and demographic predictors for three species of sandpipers measured along the southwestern coast of James Bay in two time-periods: 1974–1982 and 2014–2017. Significant *p*-values are italicized and bolded (α = 0.05). Blank cells for a parameter indicate that the parameter was not included in the model for that species. For semipalmated sandpipers, the model reference group (the intercept coefficient) is adults in the historical time-period. For least and white-rumped sandpipers, the reference group is birds in the historical time-period.

### Semipalmated sandpiper sex models

**Fig 4:**
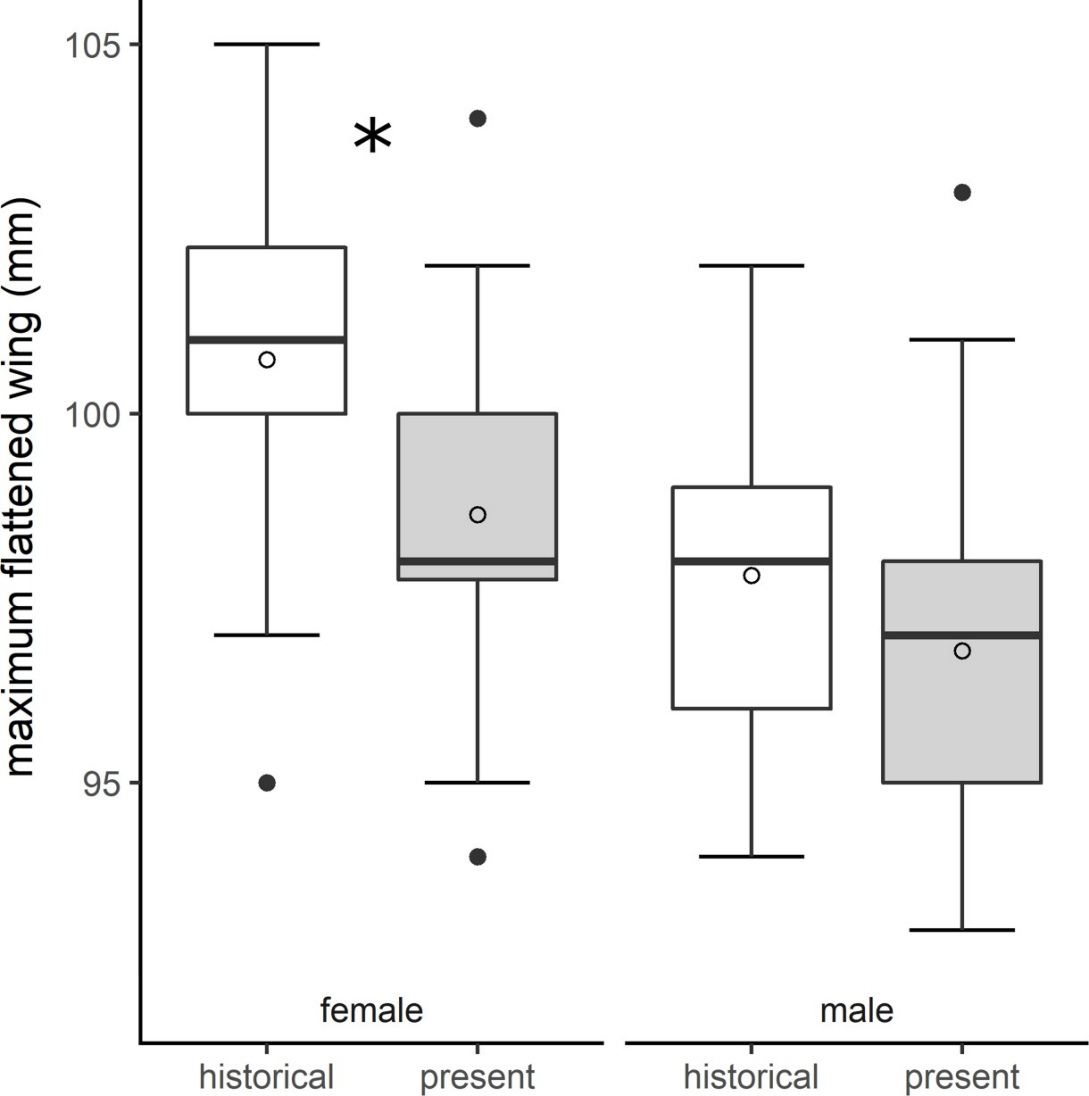
Wing lengths have shortened more for female than male semipalmated sandpipers. Historical (1974–1982) and present-day (2014–2017) differences in wing lengths (mm) of semipalmated sandpipers by sex. Significant differences are designated with * (α = 0.05).

Changes in wing length between historical and present-day semipalmated sandpipers depended on sex (F_1,220_ = 78.8, *p* < 0.001; Fig 4), and this relationship was not modified by age (F_1,220_ = 0.6, *p* = 0.45). Sample size was small for some age groups (i.e., present-day juvenile females n = 7 and present-day juvenile males n = 5). Both male and female wing lengths declined (males: 1.0 ± 0.6 mm; females: 2.1 ± 0.5 mm), but the decline was only significant for females (F_1,220_ = 5.7, *p* = 0.02). Only sex and time-period were significant predictors of bill length. Male bill lengths were shorter than female bill lengths for both age classes across time-periods (F_1,196_ = 126.5, *p* < 0.001; males: 18.4 ± 0.2; females: 20.0 ± 0.2), and bill length increased by 0.2 ± 0.3 mm between time-periods (F_1,196_ = 4.7, *p* = 0.03) for both sexes.

### Semipalmated sandpiper wing shape

**Fig 5:**
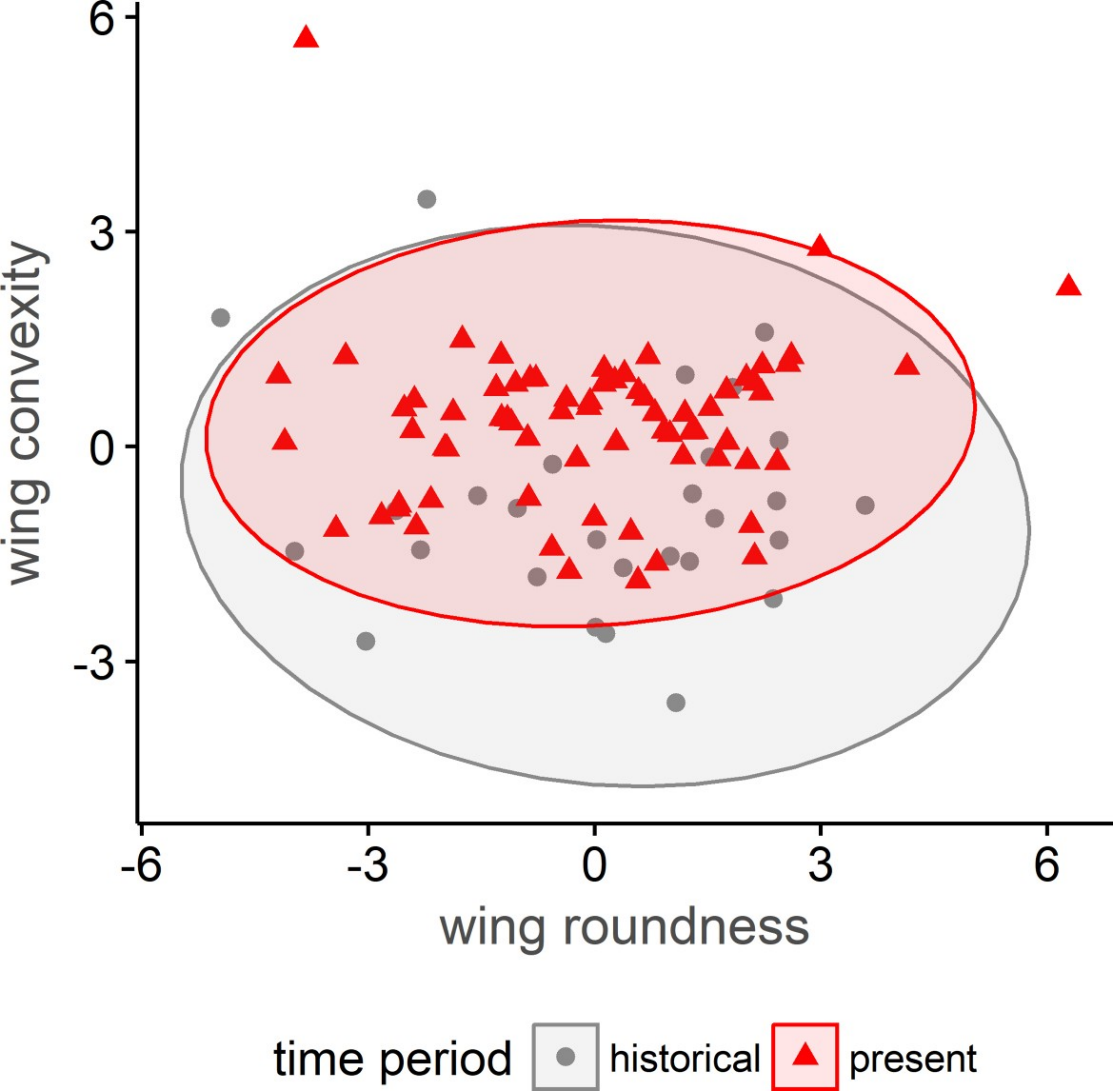
Size constrained correspondence analysis of historical and present-day wing shape of semipalmated sandpipers. Historical and present-day differences in wing shape of semipalmated sandpipers from James Bay, Ontario, Canada. Higher values on the x-axis indicate rounder wings, and higher values of wing convexity indicate wings that are more convex. Present-day birds have more convex wings than birds in the past (α = 0.05), but wing roundness has not changed.

We analyzed wings of 98 semipalmated sandpipers (historical adults: n = 17; present-day adults: n = 26; historical juveniles: n = 11; present-day juveniles: 44) for changes in shape. The first three principal components, C1, C2, and C3, explained 98% of the variance between samples (S1 Table). There was no difference between historical and present-day semipalmated sandpiper wing roundness (C2, F_1,93_ = 0.3, *p* = 0.86) and no model predictors were significant. Wing convexity, however, increased (C3, F_1,93_ = 21.8, *p* < 0.001; Fig 5) between the historical and present-day time periods, which means that the feathers closest to the longest primary in present-day birds changed less rapidly in length (i.e., broader wing tip). There was a significant effect of age in the wing convexity model (F_1,93_ = 5.0, *p* = 0.03). Adult wings were more convex than juveniles, but this difference between age groups did not change between the time-periods (F_1,93_ = 1.0, *p* = 0.32).

### Wing and bill allometry

**Fig 6:**
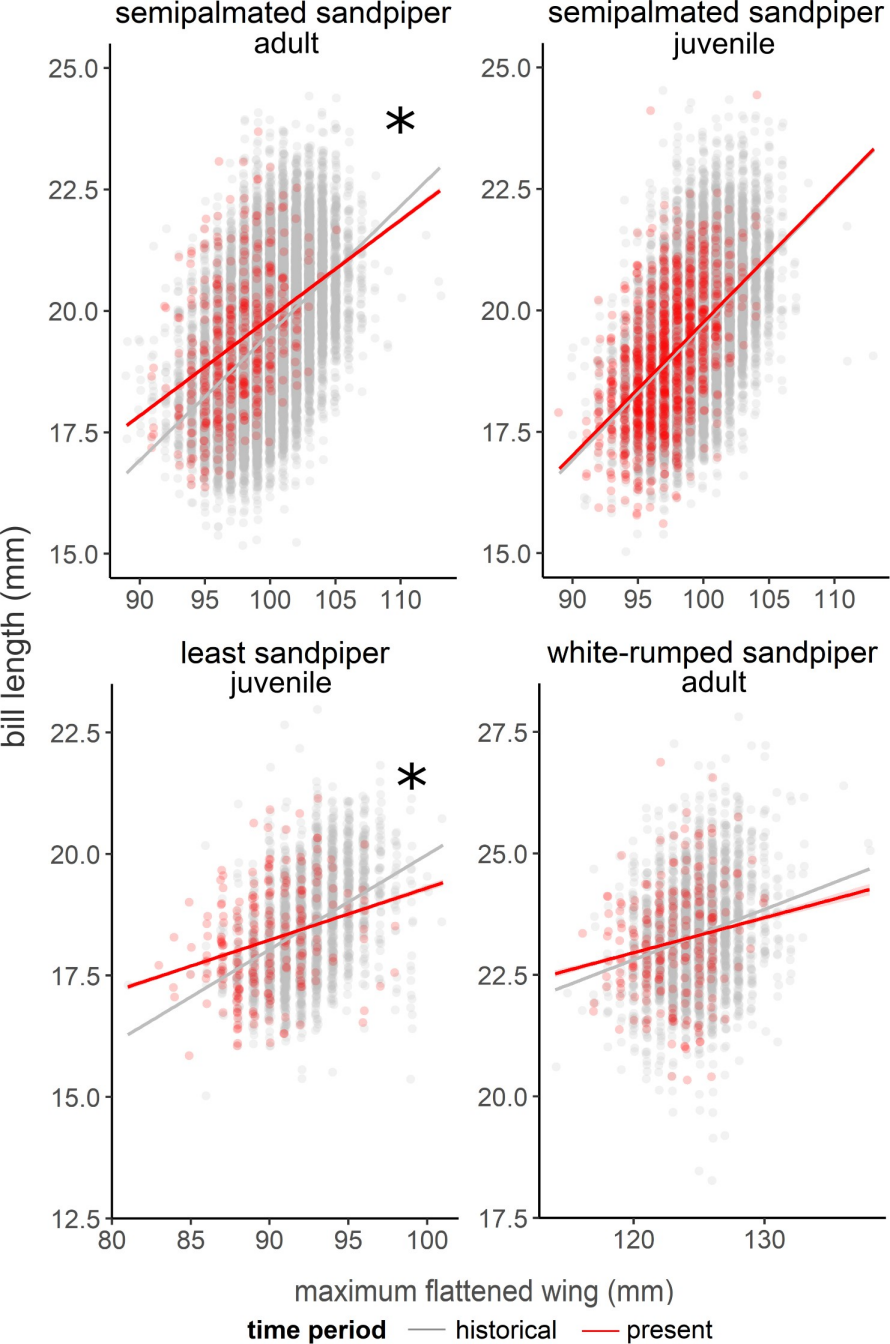
Wing and bill length allometric relationships during historical and present-day time-periods. Historical and present-day differences in covariance of wing and bill lengths of focal species and age classes of sandpipers measured at James Bay. Significant differences in present-day and historical slopes were detected for least sandpiper juveniles and semipalmated sandpiper adults (α = 0.05).

Wing and bill allometry changed for most species and age groups but not for semipalmated sandpiper juveniles (F_1,15707_ = 0.0, *p* = 0.90; Fig 6). For this group, wing length declines were paired with the same magnitude of decline in bill length that would be expected from the historical relationship (Fig 6). The relationship between wing and bill lengths for semipalmated sandpiper adult and least sandpiper juvenile birds, however, was different between time-periods (i.e., significant time-period by wing length interaction; SESA adult: F_1,24295_ = 6.6, *p* = 0.01; LESA juvenile: F_1,2516_ = 14.4, *p* < 0.001). For both groups, present-day birds have shorter wings now, for a given bill length, than in the past (Fig 6). Though white-rumped sandpipers followed this trend, the relationship was not significant (F_1,2869_ = 1.2, *p* = 0.28).

### Measurement error

Both wing length and bill length measurements had high repeatability across banders (*R* = 0.78 and *R* = 0.87 respectively, S2 Table). Although the observed mean differences in wing and bill lengths between the historical and present-day time-period fall within the repeatability coefficient for measurement error (CR; S2 Table), the mean wing length difference in measurements between observers (1.06 mm) is lower than the observed differences in wing length between historical and present-day birds (Table 2). This was not true for bill length differences; the 0.39 mm mean difference between banders for bill length is greater than the observed differences between historical and present-day birds, except semipalmated sandpiper juveniles (Table 2). The standard error of measurement (69% of repeated measurements) for both morphometrics (S2 Table) also follows this pattern.

## Discussion

This study shows consistent patterns of wing length decline between the 1980s and the present-day but inconsistent patterns of bill length change across three species of Calidridine sandpipers during stopover on southbound migration through a major subarctic stopover site, James Bay. Wing lengths declined for all species and age classes and for both male and female semipalmated sandpipers, although the relationship was not significant for males. The magnitude of declines in wing lengths for each species and age class were greater than the mean difference in measurement and standard error of measurement for small sandpipers in our historical dataset; thus, declines in wing lengths observed were unlikely to be a result of measurement error. Many banders (> 15 per time-period) were involved in measuring flattened wing cord using the same methods in the historical and present-day datasets, which limits the likelihood of measurement bias from banders that tend to measure high or low. Our results, thus, provide strong evidence of a decline in wing length for all species. In contrast, bill length declines only were significant in two groups (least and semipalmated sandpiper juveniles), and for least sandpipers, the magnitude of decline (0.2 mm) was within the mean difference of measurement and standard error, providing little evidence for bill length declines. Only bill length declines for juvenile semipalmated sandpipers were within the mean difference in measurement but outside of the measurement’s standard error, so bill lengths may have become shorter in this group.

We outline the support for our hypotheses below and in Table 4. Overall, we found little to no support for apparent change leading to patterns of wing length declines in Calidridine sandpipers, such as demographic shifts. We found limited support for geographic subpopulation shifts for semipalmated and white-rumped sandpipers because wing and bill length did not change together, except for juvenile semipalmated sandpipers. In this group, it is possible that a greater proportion of the smaller, western breeding population of juveniles are using James Bay during stopover. Though identifying breeding origin of birds at stopover sites is difficult, our results are consistent with Lank et al. [25] who reported shorter wing lengths but not bill lengths across subpopulations of semipalmated sandpipers during this time-period. When considered in concert, these results do not support the hypothesis that shorter wing lengths are a result of a shift in the relative abundance of individuals from variously sized subpopulations.

**Table 4:** Evidence for hypotheses by species and age classes.

	hypotheses						
	apparent morphometric change				true morphometric change		
	regional subpopulation shifts	changes in age proportions	changes in sex proportions	feather wear	body shrinkage	wing only shrinkage	wing shape change
**least sandpiper** **juvenile**; not tested for adults because few captured or observed at the site	*Not tested*: no knowledge of hatching location; no known geographic trend in size	*None*: juveniles are by far the dominant age class and showed shorter wings over time	*None*: no changes in slope of seasonal decline in wing and bill lengths	*None*: wing lengths of juveniles have declined	*Limited evidence*: wing and bill lengths have declined, but bill length is in the range of measurement error	*Support*: decline in wing length without decline in bill length that cannot be attributed to measurement error	*Some support*: shorter wings tend to be rounder and more maneuverable [38]; We have no direct measurements of wing shape for least sandpipers
**semipalmated sandpiper** **juvenile and** **adult**	*None*: shorter wings but not bills for adults & Lank et al. [25]; *Some support*: shorter bills and wings of juveniles could indicate an increased proportion of smaller western hatched birds	*None*: shorter wings for both age classes	*None*: no changes in slope of seasonal decline in wing and bill lengths; *Limited evidence*: female wing lengths have declined but trend of decline for males was not significant	*None*: wing lengths of juveniles have declined	*None for adults*: wing lengths have declined without change in bill; *Support for juveniles*: wing and bills declined together	*Support for adults*: declines in wing lengths without declines in bill length; *Limited evidence*: wing length and bill lengths decline together	*Support*: wings have become more convex in addition to shorter (i.e., more maneuverable)
**white-rumped sandpiper** **adult;** not tested for juveniles because few captured or observed at the site	*None*: Wing lengths but not bill lengths of adults have declined	*None*: adults are by far the dominant age class and showed shorter wings over time	*None*: no changes in slope of seasonal increase in wing and bill lengths	*None*: wing lengths of adults have declined	*None*: wing lengths have declined without change in bill	*Support*: decline in wing length without decline in bill length	*Some support*: shorter wings tend to be rounder and more maneuverable [38]

Similarly, we found little support for the hypothesis that shorter wings are the result of smaller body sizes. Wing lengths were shorter across all groups, but bill lengths were not (semipalmated sandpiper adults, white-rumped sandpipers) or could be attributed to measurement error (e.g., least sandpipers, which had a smaller bill length decline than expected for the magnitude of wing length decline). Although the effect size is small, we cannot rule out the possibility of full body size shrinkage for juvenile semipalmated sandpipers because both wing and bill lengths declined significantly and according to the historical scaling relationship expected for that species. If this effect is true, shrinkage of juveniles could be a result of nutritional stress in years of earlier snow melt [15] or an increase in the proportion of smaller birds from western breeding areas [44], [45].

In addition to age, we found that analyzing morphometric patterns by sex provided new insights into mechanisms underlying shorter wing lengths in sandpipers. Female semipalmated sandpiper wing lengths declined, but the pattern of decline for male wing lengths was not significant. In fact, present-day wings of females are approximately the same length of the wings of males during the historical period, a considerable change in a short period of time. Although sample sizes were comparatively low for these analyses, this pattern could be a result of sex-specific differences in selection for wing morphology. In some *Calidris* species, males have evolved shorter, rounder wings [38] than females, and smaller body size in males can increase acrobatic aerial display performance during courtship [40], [75]. Therefore, males have wing shapes better suited for maneuverability than females. If selection favors maneuverable wings (for example, to avoid predation by raptors), long-winged females may be less likely to survive than short-winged females or males that already have short, round wings. Consequently, selection pressure for maneuverable wings may have been stronger for females than for males and could have led to this pattern.

As expected, seasonal trends in wing lengths were detected: a seasonal decline in wing length for semipalmated sandpipers reflected the later arrival of males (shorter-winged) and a seasonal increase for white-rumped sandpipers reflected the later arrival of females (longer-winged). However, we found no evidence that these seasonal relationships have changed between time-periods. This result, combined with the finding that present-day vs historical absolute wing lengths are shorter, does not support the hypothesis of an increase in the proportion of shorter-winged males at the stopover site.

Sandpiper wings could have shortened from increased feather wear, for example, because of shifting non-breeding ranges and migrations that have become more challenging such as longer migration routes or increase predator evasion flights. Our results do not support a hypothesis of increased feather wear because wing lengths were shorter for both juvenile and adult birds, a pattern that would not be expected in juveniles because they grew their primary feathers only weeks prior to arriving at James Bay and have not undergone a complete migration at the time of stopover. In fact, the magnitude of wing length decline in adult and juvenile semipalmated sandpipers was the same (~2.0 mm decline) despite differences in timing of primary growth and migration distance. Additionally, the observed magnitude of wing length decline for juveniles (2.0% of total wing length for SESA, 2.4% of total wing length for LESA in approximately one month since feather growth) is substantially greater than the expected rate of feather wear in shorebirds (0.003–0.48% per month [51]). Thus, although the topic of feather wear warrants further study, we found no evidence to suggest that it plays an important role in the wing length declines observed here.

Predation risk was hypothesized by Lank et al. [25] as a selective force resulting in shorter, more maneuverable wings. Predation risk is an important selective pressure underlying many elements of the life history of migratory shorebirds, from the selection of stopover sites that balance foraging opportunities against predation risk [76], [77] to the decision to migrate at all [78]. The need to balance flight efficiency (long, pointed wings) with maneuverability to evade predators (short, rounded wings) presumably also has influenced the evolution of wing shape for sandpipers over evolutionary time. Even over the much shorter time scale of our study, a dramatic increase in the risk of predation by raptors (abundance increasing since the ban of DDT) may have contributed to observed changes in wing length and shape. Birds with shorter, more convex wings, such as those observed in present-day semipalmated sandpipers, may be more difficult to catch by raptors because of improved take-off performance [37] and lateral maneuverability [29]. Although there were not historical museum specimens to test if wing shape has changed for white-rumped and least sandpipers, Fernandez and Lank [38] documented a negative correlation between wing length and roundness in western sandpipers, suggesting that shorter wings may be associated more generally, with rounder wings in shorebirds.

Whether or not predation plays an important role in wing shape, flight efficiency is an evolutionary constraint for these long-distance migrants. A study of over 130 avian species showed that long-distance migrants have higher mass-adjusted wing aspect ratios (i.e., longer, more pointed wings) than short-distance migrants, and this shape minimizes the energetic cost of migration and maximizes flight speeds [79]. Shorter migration distances could reduce selection for high aspect ratio wings; however, migration distances are expected to increase for long-distance migrants under scenarios of global climate change [80–82]. Moreover, despite studies showing northward shifts of shorebird distributions in Europe [83], [84], we know of no evidence for large-scale shifts in breeding or non-breeding distributions of shorebirds across the western hemisphere [85] that would reduce the constraint for efficient, long-distance migratory flight.

Across the three species we studied, the magnitude of wing length decline follows a pattern we might predict based on the relative need for long-distance migration flight efficiency. The white-rumped sandpiper, which migrates to southern South America [86] (approximately 11,000 km from James Bay), had the smallest decline in wing length. The magnitude of decline was intermediate for semipalmated sandpipers, which migrate to Central America and northern and central South America [87], approximately 6,000 km from James Bay. It was largest for least sandpipers, which migrate to the southeastern United States, central America, and northern South America [47], approximately 4,000 km from James Bay. This gradient of wing length decline could reflect higher selective pressure for flight efficiency in longer-distance migrants. Moreover, this result provides support for the hypothesis of Møller et al. [88] that selection for energetically efficient migration may limit phenotypic and microevolutionary responses to environmental change. These results are consistent with other studies suggesting long-distance migrants are less flexible in response to environmental change [89], [90]. Nevertheless, our study found evidence of change in morphology for even extreme long-distance migrants, consistent with the hypothesis that the evolution of migration (and associated morphological traits) is labile and can occur quickly [91], [92].

Our results show that wings have become shorter in both juvenile and adult semipalmated sandpipers at the same magnitude (2.0 mm), thus rejecting the hypothesis wing length changes are the result of differences in the proportions of age classes migrating through James Bay. We also found a similar pattern of wing length decline in sympatric white-rumped and least sandpipers. Given the hemisphere-wide distribution of semipalmated, white-rumped, and least sandpipers throughout the annual cycle [47], [86], [87], the consistent pattern of wing length decline but inconsistent pattern of bill length decline observed in this study suggests a morphometric response to a wide-reaching environmental change. Our finding that shorter wings are also more convex supports the hypothesis that selection has resulted in more maneuverable wings. Studies directly linking shorebird wing shape and size to predation risk and survival, such as mark recapture analyses, would provide direct evidence to support this predation risk hypothesis. Given we found some evidence for smaller body size in juvenile semipalmated sandpipers, research on growth, development, and survival of shorebirds at different Arctic breeding sites could clarify the role of nutritional stress and climate change in development of shorter, more convex wing morphology in sandpipers.

## Supporting information

S1 Appendix Wing photo and caliper measurement calibration. Methods, model results, and calibration equations comparing shorebird wing photos to wing measurements taken with calipers.
(DOCX)

S1 Table Results from size constrained correspondence analysis (SCCA) of semipalmated sandpiper primary feathers. (DOCX)

S2 Table Measurement error results from within-year historical recaptures by different banders along James Bay, Ontario, Canada (1974–1982). (DOCX)
