# Supplementary material for: Consistent declines in wing lengths of Calidridine sandpipers suggest a rapid morphometric response to environmental change
Source: PLoS One. 2019 Apr 3;14(4):e0213930. doi: 10.1371/journal.pone.0213930 (PMC6447156; doi:10.1371/journal.pone.0213930)
Supplement: S1 Appendix — Methods, model results, and calibration equations comparing shorebird wing photos to wing measurements taken with calipers. (DOCX) [file pone.0213930.s001.docx]

**S1 Appendix. Wing photo and caliper measurement calibration.**

We used a linear mixed effects model to determine calibration equations for measurement variation between caliper measurements and photos on semipalmated sandpiper wings (n = 20). The full model tested if feather length measured by calipers could be explained by predictors of feather length measured by photo, the particular feather distance measured (∆Q_2_-∆Q_8_), and an interaction between photo measurement and the particular feather distance measured. A random effect of individual bird was included in the model to account for repeated measures on the same birds. Because P10 is usually the longest primary in semipalmated sandpipers, ∆Q_1_ was excluded from the analysis (i.e. ∆Q_1_ = 0 in both photo and calibration measurements). In instances where P9 was the longest primary (resulting in ∆Q_2_ = 0), the calibration equation for delta ∆Q_2_ was applied to ∆Q_1._

The interaction effect between photo measurement and feather measured was not significant (*p* > 0.05), so it was removed from the predictive model. Results from the final model are presented in Table A. The parameter estimate for the effect of photo measurement was 0.332 ± 0.1, resulting in a calibration equation of y = intercept + 0.332x; marginal *R^2^* = 0.99 (marginal *R^2^* is the model variance explained by the fixed effects; Nakagawa and Schielzeth 2013). Calibration equations for each measurement (∆Q_2_-∆Q_8_) are listed in Table B. Overall, for ∆Q_4_-∆Q_8_ (primaries most proximal to the body), photos tended to overestimate measurements (mean differences 0.2 – 0.9 mm), whereas photos underestimated the length of ∆Q_2_ (mean difference < 0.1 mm) and ∆Q_3_ (mean difference < 0.1 mm). Primary lengths (Q_1_-Q_8_ corresponding to P10-P3) were calculated by subtracting the ∆Q values from the longest primary length.

**Table A. Analysis of deviance table for linear mixed effects model for photo and caliper measurement calibration.**

|  | **df** | **χ^2^** | ***p*** |
| --- | --- | --- | --- |
| photo measurement | 1 | 20.1 | ***<0.0001*** |
| feather distance measured | 6 | 78.2 | ***<0.0001*** |

Significant *p*-values are bolded and italicized at (α = 0.05). Individual bird was a random factor in this model.

**Table B. Calibration equations for wing shape metrics.**

| **Distance measured** | **calibration equation** |
| --- | --- |
| ∆Q_2_ | *y* = 0.429113 + 0.331977*x* |
| ∆Q_3_ | *y* = 2.824159 + 0.331977*x* |
| ∆Q_4_ | *y* = 7.525708 + 0.331977*x* |
| ∆Q_5_ | *y* = 12.50088 + 0.331977*x* |
| ∆Q_6_ | *y* = 17.22108 + 0.331977*x* |
| ∆Q_7_ | *y* = 21.65815 + 0.331977*x* |
| ∆Q_8_ | *y* = 25.63647 + 0.331977*x* |

Calibration equations to correct measurements for each feather measured (∆Q2-∆Q8). y is the calibrated measurement equivalent to a caliper measurement, and x is the ∆Q measurement obtained from wing photos.

**References**

Nakagawa, S., & Schielzeth, H. (2013). A general and simple method for obtaining R2 from generalized linear mixed‐effects models. *Methods in Ecology and Evolution*, 4(2), 133-142.
