# Supplementary material for: Consistent declines in wing lengths of Calidridine sandpipers suggest a rapid morphometric response to environmental change
Source: PLoS One. 2019 Apr 3;14(4):e0213930. doi: 10.1371/journal.pone.0213930 (PMC6447156; doi:10.1371/journal.pone.0213930)
Supplement: S1 Table — (DOCX) [file pone.0213930.s002.docx]

**S1 Table. Results from size constrained correspondence analysis (SCCA) of semipalmated sandpiper primary feathers.**

|  | C1 | C2 | C3 |
| --- | --- | --- | --- |
| Q_1_ | 0.350 | 0.428 | -0.317 |
| Q_2_ | 0.359 | 0.359 | -0.155 |
| Q_3_ | 0.362 | 0.292 | 0.038 |
| Q_4_ | 0.362 | 0.161 | 0.212 |
| Q_5_ | 0.364 | -0.001 | 0.227 |
| Q_6_ | 0.343 | -0.309 | 0.646 |
| Q_7_ | 0.351 | -0.458 | -0.078 |
| Q_8_ | 0.337 | -0.521 | -0.595 |
| Proportion of variance explained | 91.1% | 4.6% | 2.2% |
| Cumulative proportion of variance | 91.1% | 95.7% | 98.0% |
| Proportion of shape variance explained |  | 53.4% | 22.8% |

SCCA of feather lengths (Q_1_-Q_8_ corresponding to P10-P3) of semipalmated sandpipers from James Bay, Ontario, Canada. C1 represents body size, C2 wing pointedness, and C3 wing convexity.
