# Supplementary material for: Consistent declines in wing lengths of Calidridine sandpipers suggest a rapid morphometric response to environmental change
Source: PLoS One. 2019 Apr 3;14(4):e0213930. doi: 10.1371/journal.pone.0213930 (PMC6447156; doi:10.1371/journal.pone.0213930)
Supplement: S2 Table — (DOCX) [file pone.0213930.s003.docx]

**S2 Table. Measurement error results from within-year historical recaptures by different banders along James Bay, Ontario (1974-1982).**

| measurement error | maximum flattened wing length (mm) | bill length (mm) |
| --- | --- | --- |
| n unique banders | 18 | 20 |
| n unique birds | 6398 | 3628 |
| n obs | 6398 | 6443 |
| mean difference between obs | 1.06 | 0.39 |
| *R* | 0.78 ± 0.02 | 0.87 ± 0.01 |
| BSV | 4.92 | 1.48 |
| BBV | 0.30 | 0.02 |
| WSV | 1.07 | 0.19 |
| SEM (√WSV) | 1.03 | 0.44 |
| CR | ± 2.88 | ± 1.22 |

*R* = repeatability of the measurement, BSV = between subjects variance, BBV = between bander variance, WSV = within subject variance, SEM = standard error of measurement, CR = repeatability coefficient for measurement error. The CR indicates that 95% of measurement differences on the same bird will fall within these limits while accounting for bander specific bias. The SEM indicates that 69% of measurement differences will fall within this range. All measurements are in mm.
